# Cost effectiveness of chest pain unit care in the NHS

**DOI:** 10.1186/1472-6963-8-174

**Published:** 2008-08-13

**Authors:** Yemi Oluboyede, Steve Goodacre, Allan Wailoo, the ESCAPE Research Team

**Affiliations:** 1 Health Economics and Decision Science (HEDS), School of health and related research, University of Sheffield, 30 Regent Street, Sheffield, UK; 2 Emergency Department, Northern General Hospital, Herries Road, Sheffield, UK

## Abstract

**Background:**

Acute chest pain is responsible for approximately 700,000 patient attendances per year at emergency departments in England and Wales. A single centre study of selected patients suggested that chest pain unit (CPU) care could be less costly and more effective than routine care for these patients, although a more recent multi-centre study cast doubt on the generalisability of these findings.

**Methods:**

Our economic evaluation involved modelling data from the ESCAPE multi-centre trial along with data from other sources to estimate the comparative costs and effects of CPU versus routine care. Cost effectiveness ratios (cost per QALY) were generated from our model.

**Results:**

We found that CPU compared to routine care resulted in a non-significant increase in effectiveness of 0.0075 QALYs per patient and a non-significant cost decrease of £32 per patient and thus a negative incremental cost effectiveness ratio. If we are willing to pay £20,000 for an additional QALY then there is a 70% probability that CPU care will be considered cost-effective.

**Conclusion:**

Our analysis shows that CPU care is likely to be slightly more effective and less expensive than routine care, however, these estimates are surrounded by a substantial amount of uncertainty. We cannot reliably conclude that establishing CPU care will represent a cost-effective use of health service resources given the substantial amount of investment it would require.

## Background

Acute chest pain is responsible for approximately 700,000 patient attendances per year at emergency departments in England and Wales and 20–30% of emergency medical admissions [1]. The Chest Pain Unit (CPU) has been developed to improve care and reduce costs for patients with acute chest pain. A single centre study [2] has shown that CPUs care can reduce admissions, reattendances and outpatient follow-up, and improve quality of life for selected low-risk patients who are suitable for CPU care.

The ESCAPE (Effectiveness and Safety of Chest pain Assessment to Prevent Emergency admissions) multicentre trial was undertaken to determine whether CPU could reduce admissions, improve outcomes and be cost effective across a variety of NHS hospitals. The main analysis from the ESCAPE multicentre trial [3] showed that the introduction of CPU care across a range of hospitals: 1) Did not alter the proportion of patients admitted, 2) Was associated with increased reattendances and subsequent admissions and 3) May have been associated with increased numbers of patients attending with chest pain. It concluded that establishing CPU care throughout the NHS could not be justified on the basis of any expectation of reducing emergency admissions.

Even if CPU care does not reduce hospital admissions it may still be cost-effective, compared to routine care, if it improves patient outcomes or reduces health service costs in other ways, such as by reducing length of hospital stay. We therefore planned to undertake an economic evaluation alongside the ESCAPE multicentre trial [3], using data from the trial and from external sources in a decision-analytic model (the rationale for using this type of model is explained in a later section). Our specific objective was to estimate the cost-effectiveness of CPU, compared to routine care, in terms of the incremental cost per quality adjusted life year (QALY) gained. This outcome measure allows us to combine length of life and quality of life into a single summary measure and thus allows a multidimensional comparison of CPU care with routine care.

## Methods

We developed a decision-analytic model to estimate the costs and QALYs accrued by patients with chest pain who received CPU or routine care. We assumed that CPU care could potentially influence QALYs in three ways: 1) Reducing the time delay to reperfusion for ST-elevation myocardial infarction; 2) Reducing the proportion of patients inadvertently discharged home with acute coronary syndrome; and 3) Reducing diagnostic uncertainty and thus improving quality of life.

### Data Sources

Cost and effectiveness data were split into two categories, short term or costs and effects up to 6 months, and long term or lifetime costs and effects. The economic analysis primarily used data from the ESCAPE study that provided the data for the 6 month costs and effects. This study was a cluster randomised controlled trial involving 14 hospitals – half of which implemented the CPU protocol whilst the rest provided routine care. All 14 hospitals in the pre-intervention phase provided routine care (year 1 – T1). Seven out of these were randomly selected to provide CPU care in the post-intervention phase (year 2 – T2) whilst the rest continued to provide routine care. Cost and effectiveness (EQ-5D) data were collected up to six months after initial hospital attendance using postal questionnaires mailed to a subgroup of 200 patients with chest pain before and after intervention at each hospital. Computer records were also used where appropriate to measure resource use for these patients (full details of the ESCAPE study have been reported elsewhere [3]).

#### Short term cost and effectiveness data

The following items of resource use were identified in the ESCAPE study:

1. Initial emergency department attendance, CPU care, and hospital admission.

2. Emergency department re-attendances, hospital (re)admissions, outpatient visits, diagnostic tests, operations and procedures.

3. Telephone health advice.

4. GP, nurse and social work visits.

Resource items were valued using national unit costs for 2005/2006 [4,5]. Total costs were then aggregated across patients to derive a total cost per patient up to six months. This included the additional cost of providing CPU care for all post-intervention patients attending a CPU hospital.

EQ-5D data from the ESCAPE study at one and six months was used to estimate the area under the curve for health utility. Data from all patients who attended a hospital without an active CPU (i.e. pre-intervention at CPU hospitals and both time periods at control hospitals) were used to estimate QALYs up to six months. Area under the curve data for health utility were analysed using a random effects regression model to estimate the absolute effect of availability of CPU care upon QALYs up to six months, compared to control hospitals, adjusted for baseline differences, age and gender.

#### Long term cost and effectiveness data

Long-term cost and effectiveness data for survivors with coronary heart disease were estimated from an external data source [6]. We used the same values for all patients (regardless of the underlying cause of their chest pain), because only those with myocardial infarction or ACS could potentially suffer mortality that would lead to a difference between the two strategies (CPU or routine care).

#### Other data sources

We estimated the expected mortality from ST-elevation myocardial infarction using time delay data from the ESCAPE trial and an analysis by Boersma [7] that used data from thrombolytic trial data to estimate the relationship between time delay from symptom onset to thrombolysis and expected probability of mortality for a typical patient. The Boersma equation was used to calculate expected mortality, according to their symptom onset to needle time, for all patients thrombolysed for ST-elevation myocardial infarction in the study. Data from patients attending a hospital with no active CPU (i.e. pre-intervention at CPU hospitals and both time periods at control hospitals) were used to estimate the mortality of ST-elevation myocardial infarction when no CPU is available. For the effect of CPU care upon mortality from ST-elevation myocardial infarction expected mortality data was used, calculated using the Boersma equation, to estimate the absolute effect of CPU care upon mortality. A random effects model was used to estimate the effect of CPU care compared to control hospitals, after adjusting for baseline difference between CPU and control hospitals.

In order to the estimate the effect of CPU availability upon discharge with ACS we identified all study patients who were discharged after initially attending with chest pain and then were re-admitted within 30 days with a complaint that was not obviously unrelated to chest pain. Random effects modelling was used to estimate the effect of CPU availability upon this outcome, compared to control hospitals. Adjustments were made for baseline differences between CPU and routine care in terms of age and gender. It was assumed that the relative effect of CPU availability upon this outcome was equivalent to the effect of CPU availability upon discharge with ACS. The rate of discharge with ACS at hospitals without a CPU was estimated using data from a study by Collinson et al [8] who followed up a cohort of patients with chest pain who were discharged after emergency department assessment.

Table 1 shows the parameters used to populate our model which also includes the data from external sources used in our analysis [6,8,9].

**Table 1:** Model parameter descriptions and values

Parameter	Description	Source	Distributions	
			
			Beta Distribution	
			
			Mean	Alpha (Beta)
pSTEMI	Probability of chest pain patients with ST-elevation myocardial infarction	Trial data: audited patients with ST-elevation myocardial infarction/all patients with chest pain	0.036	4800 (130000)
pACSdisc	Probability of being discharged with ACS at a hospital with no CPU	External data [8]	0.012	7.55 (621.82)
Incmort	Probability of inadvertent discharge upon ACS mortality, compared to admission	External data [9]	0.03	10.92 (353.1)

			Normal Distribution	
			
			Mean	Standard Error

mortSTEMI	Mortality of ST-elevation myocardial infarction at hospitals with no CPU	Modelled from trial onset to needle time data using Boersma equation [7]	0.1003	0.0004
CPUdisc	Effect of CPU availability upon probability of being discharged with ACS	Trial data: effect of CPU upon (re)admission of initially discharged chest pain patients	1.256	0.1962
cpu_STEMI	CPU availability impact upon mortality from ST-elevation myocardial infarction	Modelled from trial onset to needle time data using Boersma equation	0.0007	0.0013
routQALY	QALYs accrued up to six months after initial attendance at a hospital with no CPU	Trial data: Area under the curve for health utility for all patients attending a hospital with no active CPU	0.318	0.0038
cpuQALY	Effect of CPU availability upon QALYs accrued up to six months after initial attendance	Trial data: Effect of CPU availability upon area under the curve for health utility	0.0084	0.0129
ltQALY	Lifetime QALYs accrued by a typical patient with coronary heart disease	External data: Vergel et al [6]	6.829	0.3401
routCOST	Costs up to six months after initial attendance at a hospital with no CPU	Trial data: Mean cost per patient for all attending a hospital with no CPU	2405	63.5216
cpuCOST	Effect of CPU availability upon costs up to six months after initial attendance	Trial data: Effect of CPU availability upon mean cost per patient	-31	219.647
ltCOST	Lifetime costs of care for a typical patient with coronary heart disease	External data: Vergel et al [6]	10,079	2200

### Economic analysis

We developed a decision analytic model (the particular model used in this paper is a decision tree) to estimate the costs and QALYs of a population of patients attending an emergency department with acute chest pain. The model compared costs and QALYs accrued by providing CPU care to costs and QALYs accrued by providing routine care, in order to estimate the incremental cost per QALY gained by CPU care.

Decision analysis modelling was used because it allows us to estimate how a number of different potential effects of CPU care may influence a common outcome (QALYs). It also allows us to estimate the overall effect of CPU care in a way that can be compared with the effect of other health care interventions aimed at other conditions.

#### Probabilistic Sensitivity analysis (PSA)

**Figure 1:**
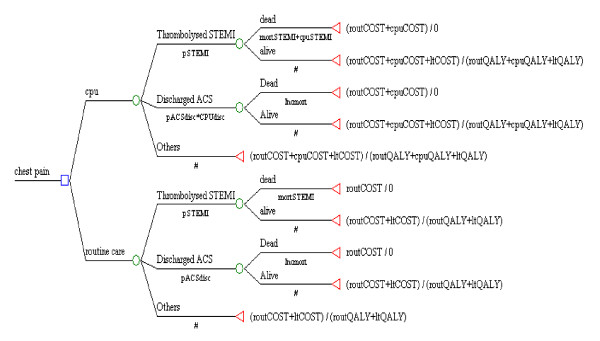
The ESCAPE decision tree comparing costs and outcomes of CPU to routine care.

Costs and outcomes were modelled using the decision tree outlined in Figure 1 to estimate the incremental cost per QALY gained. We undertook probabilistic sensitivity analysis (PSA) modelling, using Microsoft Excel. PSA is a widely used technique that allows estimation of the effect of varying model parameters in order to take account of the uncertainty in the parameter estimates that inform our cost effectiveness model. Each of the parameters we use in our model has been estimated with a degree of uncertainty and thus there is a need to spread joint parameter uncertainty to reflect the decision uncertainty. As our model is non linear the use of PSA provides the only unbiased estimate of mean cost effectiveness. Finally, the model assumes that there are no limitations in capacity, such as the availability of hospital beds and staff. However, because of the timeframe of our analysis, we feel that this is not an unrealistic assumption.

#### The Decision Tree

The decision tree outlined in Figure 1 was constructed to allow comparison of a decision to manage patients with a CPU or to use routine care. With each strategy (CPU or routine care) patients could follow one of three pathways. 1) A proportion of patients will have a ST-elevation myocardial infarction (pSTEMI) and will be eligible for thrombolysis. This proportion does not depend upon whether the hospital has a CPU or not, but may vary between individual hospitals. Of these individuals a proportion of thrombolysed patients will die within 30 days (mortSTEMI). This proportion will depend upon whether the hospital has a CPU and whether CPU care affects time delays to thrombolysis (cpu_mortSTEMI). 2) A proportion of patients with chest pain will have ACS and be inadvertently discharged without treatment (pACSdisc) [8]. This proportion will depend upon whether the hospital has a CPU and whether CPU care affects the probability of inadvertent discharge with ACS (CPUdisc). Patients who are discharged with ACS will have an increased probability of dying (Incmort), compared to those who are not discharged [10]. This increased probability of dying is not dependent upon CPU care and does not vary between hospitals. 3) All other patients who do not die and do not go through the first two pathways will have quality of life over the next six months determined by whether they attended a hospital with CPU care (cpuQALY) or not (routQALY).

Resource use costs up to 6 months after original attendance are reflected in the parameters routCOST, while cpuCOST reflects the effect of CPU availability upon costs over this time period.

We applied a beta distribution to the following probabilities p_STEMI, pACSdisc and Incmort and a normal distribution to the rest of the parameters (see Table 1). Monte Carlo simulation was used to sample from each independent parameter distribution and calculate the costs and outcomes associated with each strategy, running 1000 replications of the model.

### Model Assumptions

We assumed that CPU care could influence health service costs up to six months after attendance, and costs thereafter would be determined by whether the patient survived or not. We assumed that CPU care could influence outcomes in three possible ways:

1. Reducing death from myocardial infarction by reducing time to thrombolysis for patients with ST-elevation myocardial infarction (STEMI)

2. Reducing death from acute coronary syndrome (ACS) by reducing the proportion of patients discharged with ACS (inappropriate discharge)

3. Improving quality of life for all patients by providing a more rigorous diagnostic assessment

For all three pathways, after six months survival, we assume that quality of life and resource use will be independent of care initially received (ltQALY and ltCOST respectively). We also assumed that deaths due to other causes would not be influenced by the care initially received.

## Results

The values used to populate the economic model were:

1. The proportion of chest pain patients who had ST-elevation myocardial infarction varied across the trial hospitals from 1.6 to 7.8% with an overall mean value of 3.6% (95% CI 3.5 to 3.7).

2. Mean expected mortality among patients with ST-elevation myocardial infarction was 10.03% (95% CI 9.96 to 10.10) among patients who attended when no CPU was available.

3. The estimated effect of CPU availability upon expected mortality from ST-elevation myocardial infarction was a non-significant increase of 0.07% (95% CI -0.18 to 0.33).

4. The proportion of chest pain patients subsequently discharged with ACS at a hospital with no CPU was estimated to be 1.2% (95% CI 0.6 to 2.3) [8].

5. The odds ratio for the adjusted effect of CPU care upon the proportion of patients who were discharged and then subsequently admitted was 1.26 (95% CI 0.92 to 1.72; p = 0.146), giving a relative risk of 1.256 (95% CI 0.921 to 1.705).

6. The effect of inadvertent discharge upon mortality from ACS, compared to admission was estimated to be a 3% absolute increase using data from a literature review outlined in our previous model [9].

7. The mean area under the curve for health utility up to six months after attending a hospital with no CPU available was estimated to be 0.318 QALYs (95% CI 0.311 to 0.326) using trial data from patients attending CPU hospitals before intervention and attending a control hospital at any time.

8. The estimated effect of CPU upon mean area under the curve for health utility up to six months was a non-significant improvement of 0.0084 (95% CI -0.0168 to 0.0337; p = 0.512).

9. Discounted quality-adjusted life expectancy (after the initial six months) for patients with coronary heart disease was estimated to be 6.83 QALYs [6].

Costs per patient up to six months ranged from zero to £28,248, with a mean of £2385 and a median of £1566. Mean cost per patient was £2468 before and £2326 after intervention at the CPU hospitals, and was £2417 before and £2330 after intervention at the control hospitals. The mean cost per patient for all patients who attended a hospital with no CPU (i.e. all patients at control hospitals and pre-intervention patients at CPU hospitals) was £2405 (95% CI 2280 to 2529). The introduction of CPU care was associated with a non-significant £31 per patient reduction in 6-month costs (95% CI -400 to 461, p = 0.889).

### Cost effectiveness ratios

The model showed that CPU care was associated with a small non significant increase in effectiveness of 0.0075 QALYs per patient (95% CI -0.0168 to 0.0331), and non significant decrease in costs of £32 per patient (95% CI -480.41 to 399.86). Hence, inclusion of costs beyond six months had only a small effect on the estimated cost differences between strategies (routine or CPU care). CPU care therefore dominates in the base case analysis.

**Figure 2:**
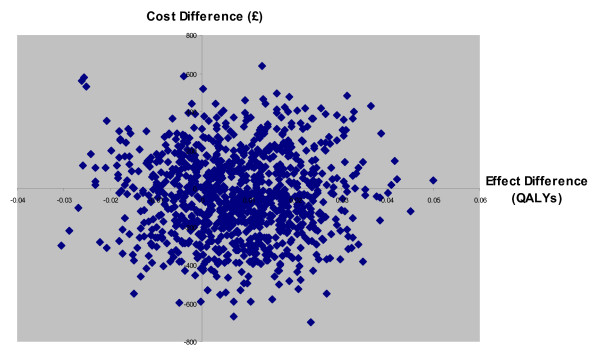
Cost-effectiveness plane for CPU compared to routine care.

Figure 2 shows the cost-effectiveness plane for CPU compared to routine care. This is a plot of the results of the PSA (for 1000 replications of the model) showing the difference in effectiveness (X-axis) and costs (Y-axis) between CPU and routine care. Each point provides an estimate of whether CPU is cost-effective compared to routine care: 408 of the estimates lie in the south-east quadrant (CPU dominates routine care), 314 lie in the north-east quadrant (CPU more effective but more expensive), 152 lie in the south-west quadrant (CPU cheaper but less effective) and 126 lie in the north-west quadrant (routine care dominates). Although the baseline estimates suggest that CPU dominates routine care, this highlights the substantial uncertainty around this point estimate, as the predicted estimates fall in all four quadrants of the cost-effectiveness plane.

**Figure 3:**
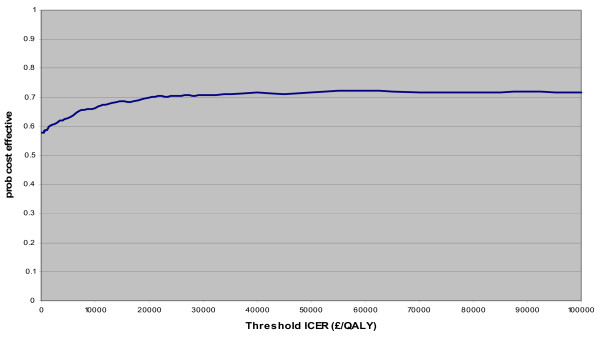
Cost-effectiveness acceptability curve for CPU compared to routine care.

Figure 3 shows the cost-effectiveness acceptability curve for CPU compared to routine care. This shows the probability that CPU will be considered cost-effective plotted against the threshold for willingness to pay per QALY gained, ranging from zero to £100,000 per QALY. If we are not willing to pay anything to gain additional QALYs, CPU care is still slightly more likely to be cost-effective than routine care (about 56% that CPU care is less costly than routine care). At a willingness to pay threshold of £20,000 for an additional QALY then the probability that CPU care will be considered cost-effective rises to about 70%. No matter how much we are willing to pay the probability of CPU care being cost-effective does not reach 75%.

## Discussion

We found that on average CPU care would result in a cost saving compared to routine care, although this was not a statistically significant result. The probability that CPU care is cost effective is approximately 70% at a willingness to pay threshold of £20,000 per QALY. This finding, however, is subject to uncertainty and it is necessary to bear in mind the substantial investment that would be needed to provide CPU care throughout the NHS. On the basis of this analysis we cannot justify widespread implementation of CPU care throughout the NHS.

Most previous cost-effectiveness analyses of CPU care originate from the United States (Roberts, Farkouh, Gomez [11-13]). These studies were based on randomised trials comparing CPU care to inpatient care that primarily compared resource use and did not evaluate patient-centred outcomes. They showed that CPU care was cheaper than routine care, but it is not clear whether this finding can be extrapolated outside the United States. It is also only relevant if CPU care replaces inpatient care. Although practice in the UK has changed recently, with increasing use of percutaneous coronary intervention, the findings of the ESCAPE trial [14] suggest that many patients with chest pain still do not receive inpatient care.

One previous cost-effectiveness analysis in the UK, based on a single-centre randomised trial [2], showed a significant improvement in QALYs and a non-significant reduction in costs among selected low risk patients who were deemed suitable for CPU care. These findings were not fully reproduced in the ESCAPE trial. Both the QALY gain and cost reduction associated with CPU care were smaller than in the single-centre trial and were non-significant. This is presumably because the ESCAPE multicentre trial evaluated the effect of CPU care across all patients with chest pain, rather than selected low-risk patients. The effect of CPU care may therefore have been lost amongst the overall population with chest pain or balanced by negative effects from implementing CPU care upon patients who attended CPU hospitals with chest pain but did not receive CPU care.

This economic analysis has an important limitation that needs to be taken into account in interpreting the findings. The comparison of the costs and effects of CPU versus routine care is made on a per patient basis, which assumes that CPU and routine care will be applied to the same population of patients attending hospital. In other words, it assumes that the decision to implement CPU care or continue with routine care does not influence the size or characteristics of the population requiring the services. However, it has been reported elsewhere [14] that the implementation of CPU care may be associated with increased attendances with chest pain and increased medical admissions. If introducing CPU care leads to increased attendances with chest pain then the assumptions in this analysis will not hold. Furthermore, whereas it is clear that additional attendances will incur health service costs, we do not have any evidence that they will gain benefit from their attendance.

Thus evidence, which cannot be incorporated in the economic model, leads us to believe that introducing CPU care may increase health service costs without any corresponding evidence that it will improve outcomes. In these circumstances it is difficult to claim that CPU is likely to be cost-effective, especially considering the substantial uncertainty surrounding the results on the economic analysis.

Another limitation is that there was considerable variation in outcomes between the individual hospitals involved in the ESCAPE trial and in the effect of introducing CPU care at individual CPU hospitals. We therefore cannot exclude the possibility that individual CPUs can markedly reduce costs and improve outcomes. Indeed, the previous single centre study [2] showed that an active CPU can improve outcomes and possibly reduce costs among selected patients. However, we can reasonably conclude that widespread implementation of CPU care is unlikely to represent a cost-effective use of health service resources.

We recently surveyed the development of chest pain services in the UK [15] and found that formal development of CPU care was limited and mostly restricted to within trials, although there was substantial informal and ad hoc development of acute chest pain services. The findings of our analysis and the ESCAPE multi-centre trial suggest that this progress is probably appropriate and that investment in formal development of CPU care throughout the UK would be misplaced.

## Conclusion

We found that introducing CPU care had no statistically significant effect on either costs or outcomes compared to routine care. Our findings are highlighted by the fact that the predicted estimates fall in all four quadrants of the cost effectiveness plane. These results do not support the widespread implementation of CPU care.

## List of abbreviations

ACS: acute coronary syndrome; CI: Confidence interval; CPU: chest pain unit; ECG: electrocardiogram; ESCAPE: Effectiveness and Safety of Chest Pain Assessment to Prevent Emergency Admission; GP: general practitioner; HRG: Healthcare Resource Group; QALY: quality-adjusted life year.

## Competing interests

The authors declare that they have no competing interests.

## Authors' contributions

All authors contributed to the data analysis, writing the article and approved the final draft. YO and SG are the guarantors.

## Pre-publication history

The pre-publication history for this paper can be accessed here:


